# Effectiveness of a stepped primary care smoking cessation intervention (ISTAPS study): design of a cluster randomised trial

**DOI:** 10.1186/1471-2458-9-48

**Published:** 2009-02-04

**Authors:** Carmen Cabezas, Carlos Martin, Silvia Granollers, Concepció Morera, Josep Lluis Ballve, Elvira Zarza, Jordi Blade, Margarida Borras, Antoni Serra, Diana Puente, Grupo ISTAPS [Intervención Sistematica sobre Tabaquismo en Atención Primaria de Salud]

**Affiliations:** 1 Departament de Salut, Generalitat de Catalunya, Spain. Institut d'Investigació en Assistència Primària Jordi Gol (IDIAP Jordi Gol), Barcelona, Spain; 2 Department de Medicina, Universitat Autònoma de Barcelona, ICS, ABS Passeig de Sant Joan, SAP Dreta Barcelona, IDIAP Jordi Gol, Barcelona, Spain; 3 Escola d'infermeria, Universitat de Barcelona, Institut Català de la Salut, Barcelona, Spain; 4 ICS. Àrea d'Avaluació, Serveis d'Atenció Primària, Àmbit Territorial Girona, Spain; 5 ICS. ABS Florida Nord, L'Hospitalet de Llobregat, Spain; 6 ICS. SAP Hospitalet. Barcelona, Barcelona, Spain; 7 ICS. ABS Jaume I, SAP Tarragona, Valls, Spain; 8 ICS. ABS Les Borges del Camp, Tarragona, Spain; 9 ICS. ABS Penedès rural, Tarragona, Spain; 10 IDIAP Jordi Gol. Barcelona, Barcelona, Spain

## Abstract

**Background:**

There is a considerable body of evidence on the effectiveness of specific interventions in individuals who wish to quit smoking. However, there are no large-scale studies testing the whole range of interventions currently recommended for helping people to give up smoking; specifically those interventions that include motivational interviews for individuals who are not interested in quitting smoking in the immediate to short term. Furthermore, many of the published studies were undertaken in specialized units or by a small group of motivated primary care centres.

The objective of the study is to evaluate the effectiveness of a stepped smoking cessation intervention based on a trans-theoretical model of change, applied to an extensive group of Primary Care Centres (PCC).

**Methods/Design:**

Cluster randomised clinical trial. Unit of randomization: basic unit of care consisting of a family physician and a nurse, both of whom care for the same population (aprox. 2000 people). Intention to treat analysis.

Study population: Smokers (n = 3024) aged 14 to 75 years consulting for any reason to PCC and who provided written informed consent to participate in the trial.

Intervention: 6-month implementation of recommendations of a Clinical Practice Guideline which includes brief motivational interviews for smokers at the precontemplation – contemplation stage, brief intervention for smokers in preparation-action who do not want help, intensive intervention with pharmacotherapy for smokers in preparation-action who want help, and reinforcing intervention in the maintenance stage.

Control group: usual care.

Outcome measures: Self-reported abstinence confirmed by exhaled air carbon monoxide concentration of ≤ 10 parts per million. Points of assessment: end of intervention period and 1 and 2 years post-intervention; continuous abstinence rate for 1 year; change in smoking cessation stage; health status measured by SF-36.

**Discussion:**

The application of a stepped intervention based on the stages of a change model is possible under real and diverse clinical practice conditions, and improves the smoking cessation success rate in smokers, besides of their intention or not to give up smoking at baseline.

**Trial Registration:**

Clinical Trials.gov Identifier: NCT00125905

## Background

The data from the 50-year follow-up study involving British male doctors showed that one in two regular smokers of cigarettes die as a result of tobacco-related disease. The latest estimations showed that in Spain in 2001 there were 54,233 deaths attributable to tobacco consumption [1]. The data of the National Health Survey [*Encuesta Nacional de Salud*] of 2006 showed that 29,9% of the adult Spanish population smoked [2], with a clear tendency towards a decrease in males and an increase in females.

Primary care can play a key role in the control of tobacco consumption. The data from our country show that in many zones more than 75% of the population attend an outpatient consultation in the local Primary-Care Centre (PCC) at least once a year, and the average number of visits varies between 5 and 6 (data from the primary-care database of 2001; Sistema de Información de Atención Primaria, 2001). Spain has an almost universal coverage of the population by a public health system in which primary care is the first point of contact. This provides a unique opportunity for intervention, and therefore prevention, in a large number of subjects on many occasions even before chronic or severe diseases have begun. Furthermore, surveys as those cited above showed that more than 60% of smokers had a desire to quit smoking, and the majority of these individuals have at some time or other made an attempt to achieve this.

In the past few years, several national and international clinical practical guidelines have been developed with recommendations to intervene on the consumption of tobacco, based on help provided at health centers, both at primary and specialist care levels.

Since 1996, various clinical guidelines integrating all the evidence available from existing clinical research have been published in different countries. Most of them were from anglosaxon countries (mainly the USA, UK and Australia). For example, the guidelines from the US Public Health services published in 2000 (recently updated)[3] were based on approximately 6000 original articles and abstracts of congresses written in English and published between 1977 and 1999. More than 50 meta-analysis were carried out and became the basis for the recommendations developed by a panel of 18 experts. Following this, there was an external revision process by another group of 70 experts [4].

The guidelines from the United Kingdom originated from the meta-analysis derived from the American guidelines and from the Cochrane international collaboration reviews [5]. Furthermore, in the year 2000, appeared the First European Directives on the Treatment of Tobacco Dependence within the European Cooperative Project of the World Health Organization.

Some of the recommendations of these guidelines have been based on a large number of clinical trials and studies of intervention. However, in other cases, there has been weak evidence and the recommendations have been based on the consensus opinions of experts.

Examples of intervention from the primary-care perspective for which there has been grade A evidence (based on many high quality clinical trials containing consistent results) are:

• Primary health care teams should ensure that their records concerning which of their patients smoke are kept up to date [A]

• GPs should advise current smokers to stop during routine consultations at least once a year, offer a prescription for Nicotine Replacement Therapy (NRT), record the response to that advice, and arrange follow up where appropriate. [A]

• At the beginning of the study, and even now, the level of evidence for some key recommendation were still C and mostly derived from published guidelines (recommendations based on consensus). These include:

▪ The role of motivational interviewing in accelerating changes in the process of smoking cessation.

▪ The role of the primary care professionals in performing more intensive interventions.

▪ The role of primary care nurses in motivating and helping patients to quit smoking.

▪ The use in primary care of non-nicotine based pharmaceutical products such as bupropion, an atypical antidepressant considered in the guidelines as a first-line drug. At the start of this study there was only limited experience of its use in primary care.

There is only partial evidence for several of these recommendations. For example, in the case of motivational interviewing [6,7], the study by Butler et al showed outcomes less positive than those expected [8], and subsequent reviews showed some inconsistencies [9-12]. Motivational interviewing is difficult to evaluate because of its intrinsic nature (variability and adaptation to characteristics both from patients and health professionals, and from the interaction between them in each clinical encounter) and also due to the difficulty of finding useful and appropiate measures of success. Another important aspect to consider is the fact that motivational interviewing requires a change in the way health professionals interact with patients.

The Cochrane review of smoking cessation interventions conducted by nurses showed that there can lead to potential benefits and that there is fair evidence of their effectiveness. Again there are few studies in primary care and some of these studies show that the interventions carried out during preventive check-ups are less effective [13].

The use of bupropion was not yet protocolised in primary care, although the Cochrane review does recommend it as a first-line drug. [14].

One of the conceptual models that has been most extensively used to explain the process of change in addictive behaviour is that developed by Prochaska and DiClemente in 1982. These researchers defined a series of successive stages each of which require different types of interventions [15,16]. The use of this model is frequently linked to the motivational interviewing strategies described earlier. It is recommended that the interventions applied in primary care must be orientated towards achieving advances in this addiction process, and not only to giving up smoking. As such, stage change can be used as an intermediate measure of the effectiveness of the interventions. The majority of the clinical guidelines used in our Spanish environment include an assessment of the stage of smoking cessation in which the individual smoker is currently located [17].

Although the amount of research about smoking intervention is impressive, many of the studies have been conducted in highly selected populations of smokers and there have not been any studies that have evaluated all the range of interventions developed, applying them in a stepped manner to non-selected smokers who consult primary care centers for other reasons. Interventions include brief motivational interviews for smokers at the precontemplation – contemplation stage, brief intervention for smokers in preparation-action who do not want help, intensive intervention with pharmacotherapy for smokers in preparation-action who want help, and reinforcing intervention in the maintenance stage.

In Spain, the first studies date from the end of the 1980s when a group of primary care professionals in Barcelona (including two of the researchers of this study; Cabezas C and Martin C) replicated the effectiveness of the brief intervention study of Russell et al in the UK in 1979 [18,19].

Since then, and extending their field of study to include the primary care environment, the same research team have evaluated the effectiveness of nicotine chewing gum and nurse-based advice [20]. Subsequent studies by Quilez, Salvador, Comas, Torrecilla, etc. [21-24] have found results consistent with those of the international studies. Some of these studies had also been carried out in primary care centers, but by health-care professionals specially trained in this area.

Among the published studies there are at least two types of interventions which have been widely evaluated: the Doctors Helping Smokers (DHS) Program proposed by Solberg [25,26], adapted to Spain by Solberg and Nebot and applied in Spain by Martin, Casas, Cordoba and Ballvé (members of the ISTAPS research group) and the Program to Quit Tobacco by Grandes and Cortada [27]. The DHS program is based on the proposals of the first guidelines of American National Cancer Institute at the end of the 1980s which are easily applied in primary care. The main limitations of the program arose from the very brief intervention proposed and from the lack of a formal evaluation of its effectiveness in Spain. [28].

The study by Grandes et al. is a quasi-experimental study which included a large number of smokers with an evidence-based strategy. However, it does not include current recommended strategies such as motivational interviewing, more intensive help (the authors evaluated a therapeutic plan with three visits and one telephone call), nor the use of other drugs (other substitutes for nicotine than patches and bupropion) [29].

Hence, when faced with reviewing the evidence and developing a clinical guideline for Spain, the authors of the present project found that there was no clear evidence either for some of the proposed recommendations, or for the overall strategy. This led to the current proposition for a study identifying this evidence. In order to put into practice the intervention proposed health professionals must be trained. Some parts of the intervention (those related with motivational interviewing) require not only the development of skills but also a change in the way health professionals interact with patients. For this reason, and in order to minimize contamination, the unit of randomization and analysis was the basic care unit consisting of a physician and nurse who take care of the same list of patients, both of whom received a 20-hours training course at the beginning of the study.

The opportunity to detect and follow-up a cohort of persons from before quitting smoking to more than 1 years post-quitting would facilitate the study of variation in the overall health-related quality of life produced as a result of the process of quitting smoking, and also provide a measure of the impact on health status. The studies published about this theme are comparisons of the perceptions of health-related quality of life in smokers and ex-smokers [30]. Serial data in clinically relevant moments of the smoking cessation process would highlight the times and the scale at which the most relevant changes in the health-related quality of life are produced.

The SF-36 is a multi-purpose, short-form health survey validated in Spain by Alonso et al [31]. It consists of 36 questions measured in an ordinal scale. It can be self-administered or used in personal or telephone interviews. This yields a profile of functional health and well-being score, as well as psychometrically-based physical and mental health summary measures The profile considers 8 scales: Physical Functioning, Role-Physical, Bodily Pain, General Health, Vitality, Social Functioning, Role-Emotional, Mental Health and two summary measures: Physical Component Summary (PCS) and Mental Component Summary (MCS).

Additionally, one of the items evaluates the change in health status over the previous year. The items detect not only positive but also negative health status. The SF-36 is sufficiently sensitive to changes when compared to other questionnaires such as the Nothingham or the Sickness Impact Profile that have been especially useful in clinical trials up until now. One version of the SF36 that measures the health perception over the precedent week, known as the acute version, enables the changes to be evaluated within the interval of time envisaged in the present study.

## Objectives

### Objectives

• To evaluate the effectiveness of a stepped smoking cessation intervention based on a transtheoretical model of change that uses the pharmacological and non-pharmacological methods proposed by evidence based Clinical Practice Guidelines for smoking cessation in primary care centres. The effectiveness will be measured at patient level as the continuous abstinence for at least one year, adjusting for the effect of cluster randomization.

• To assess the health-related quality of life change in relation to the smoking cessation process.

## Methods/design

### Study design

Cluster randomized clinical trial

### Setting

Primary Care Centers (PCC).

### Study population

All the basic care units of health professionals belonging to the Spanish Preventive Services and Health Promotion Research Network (redIAPP) that expressed their intention to participate were included (n = 121). These health professionals worked in 82 primary care centres in 11 different regions of Spain.

Between the years 2003 and 2004, individuals aged between 14 and 75 years old who attended the primay care center for any reason, who answered positively to the question "Do you smoke?" and who provided informed consent to participation in the present study, were recruited. People suffering from terminal illnesses, severe psychiatric disorders, addiction to other psychoactive substances, or those unwilling to participate in the study were excluded. The recruitement continued for 12 months.

Unit of Randomization: Basic care unit. The system of randomization was centralized and computer generated.

### Description of the intervention

The intervention consisted in the application of the recommendations of a evidence-based practical clinical guideline developed over 6 months by a team from the research group. The guideline was prepared for the Primary Care Division of the Catalan Institute of Health and which has not yet been distributed.

The basic steps in the intervention are:

1. All individuals accessing the PCC, once the motivating problem of the clinical visit had been resolved, were asked about tobacco consumption, their willingness to quit smoking (the stage of change according to the model defined by Prochaska and DiClemente) and their need for specific help in quitting smoking.

2. Individuals in the pre-contemplation or the contemplation stage were administered a brief motivational interview based on the Rollnick and Butler [8] model and were provided with a leaflet containing motivational information.

3. Individuals in the preparation or action stage who preferred the option of no specific help, received a minimal intervention which included brief advice, a leaflet containing practical information on how to quit, an offer/prescription of nicotine substitute if their nicotine dependence was medium or high, and one follow-up clinical visit or telephone call.

4. Individuals in the preparation or the action stage who requested specific help, received an intensive intervention comprising of 9 scheduled follow-up visits over 1 year wich included behavioral intervention, and use of pharmacological agents (substitutes for nicotine or bupropion according to the characteristics of the subject and their previous experiences as regard quitting smoking). They were provided with a leaflet of practical information on how to quit and another with specif advice on how to use the pharmacological treatment.

5. Individuals in the maintenance stage were provided with reinforcement advice.

All the individuals included in the intervention group had the intervention applied in a period of 6-months after their inclusion in the study. In the case of scheduled visits for smoking cessation and non-attendance, the motive was sought out. The successive interventions were adapted to each person's evolution according to the Prochaska and DiClemente's Stages of Change Model.

All the health care professionals in the intervention group attended a 20 hours course that uses techniques such as role playing, as well as a training session in the practical aspects of the protocol.

Control: The patients in the control group received the usual care in the PCC.

### Outcome measures

Self reported abstinence confirmed by a breath carbon monoxide concentration of 10 parts per millions or less, calculating point prevalence at the end of intervention, then at 1 and 2 years after the beginning of intervention, and continuous abstinence rate for 1 year.

The measurement of breath carbon monoxide was carried out by trained health professionals using Bedfont Scientific Ltd. Smokerlyzers following a pre-established protocol.

Other outcome measures:

• Change of stage in the Prochaska and DiClemente's Stages of Change Model.

• Health-related quality-of-life (HRQoL): Medical Outcomes Study 36-Item Short-Form Health Survey (SF-36) acute version (1-week recall period) in persons who quit smoking at baseline and at 2, 4, 12, 26, 52 and 104 weeks after having quit smoking.

### Data collection

An ad-hoc questionnaire that included data relating to:

• Socio-demographic characteristics (age, gender, educational level, social class according to the classification of the UK Registrar General's social classification (RGSC)).

• Anthropometric data (weight, height, blood pressure).

• Characteristics of individual's tobacco consumption (daily consumption in cigarettes/day, years of smoking, age at the start, time before first cigarette of the day, nicotine dependence measured by the Fagerström test, motivation level measured by the Richmond test), help sought to quit smoking, perceived support or lack from family, friends and co-workers.

• Variables related to the Prochaska – Diclemente transtheoretical model of change: stages, self-change processes, self-efficacy and decisional balance.

• Readiness and willingness to quit (perception of importance, confidence and preparation to quit; Richmond test).

• Morbidity (whether or not related to tobacco consumption) and intensity of use of primary care services.

• Alcohol consumption, use of other recreational drugs and physical exercise.

• Variables related to the smoking intervention provided:

▪ Professional who undertook the intervention (physician or nurse)

▪ Pharmacological treatment used: type, timetable, side effects

• As regards people who managed to give-up smoking:

▪ Ratings of tobacco abstinence symptoms (urges to smoke, irritability/frustration/anger, anxiousness, difficulty concentrating, restlessness, hunger, impatient, craving for cigarette/nicotine, drowsiness, depression/feeling blue, and desire for sweets)

▪ Scale of anxiety and depression (Goldberg test)

▪ MOS-SF-36

This questionnaire was divided in two parts (one clinical and another non clinical) and administered at baseline, at the end of intervention (6 months), and after one and two years from the start. The morbidity and use of services data were obtained by health professionals from clinical records.

In people who gave-up smoking, the specific questions related to the quitting process were assessed again in extra interviews at week 2, and at 1, 2 and 4 months after quitting smoking.

The clinical part of the questionnaire was administered in primary care centers by the health professional involved in the study. The non clinical part was administered by phone by especially trained interviewers who were blinded to the patient group assignment.

### Follow-up period

Two years from the start of the intervention.

**Figure 1:**
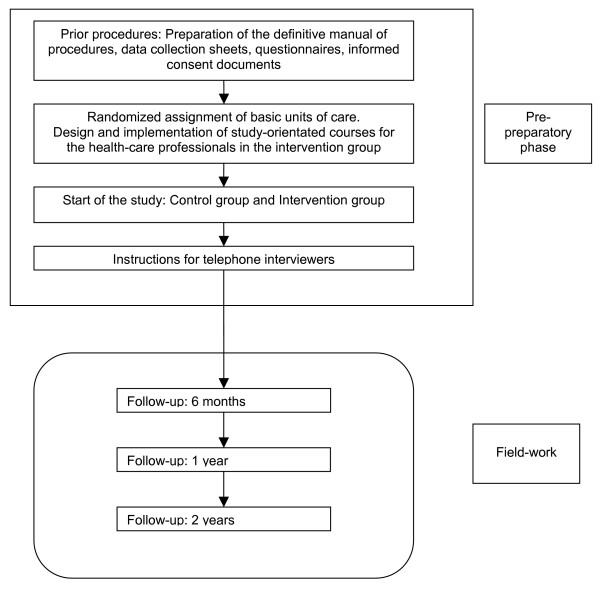
**Time-line of the ISTAPS project**.

**Figure 2:**
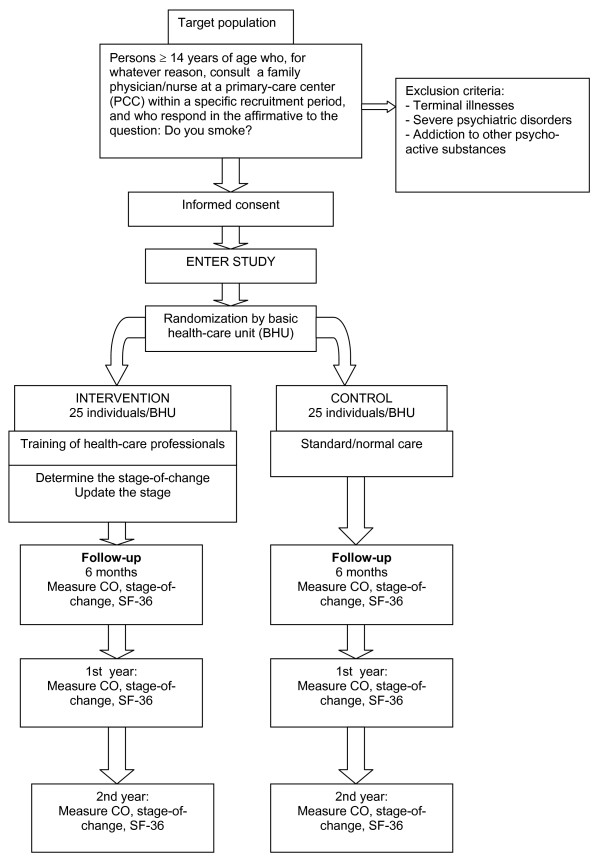
**Algorithm of the study**.

Figures 1 and 2 shows: Time-line of the ISTAPS project and ALGORITHM OF THE STUDY.

### Sample size calculation

Based on different assumptions

• Power 80%, alpha 0.05, potential study withdrawal (15%).

• A difference of 5% between the success rate of the intervention (10%) and control group (5%) -> a sample size of 499 in every group was needed.

• A difference of 20% between the success rate of the intervention (30%) and control group (10%) for people in the preparation stage -> a sample size of 110 in the preparation stage in every group was needed. Previous studies in Spain (Grandes et al. 2001) showed that approximately 16% of smokers seen in primary care centres were in the preparation stage, and thus, it was necessary to include 687 smokers in every group to obtain 110 in the preparation stage. As this number is greater than the 499 stated previously, this number is used for input in adjusting for the design effect.

• It was considered feasible that each basic care unit would include some 25 individuals in the study.

• The intracluster correlation coefficients (ICC) for outcome variables in cluster randomization clinical trials on guidelines implementation strategies in primary care are generally below 0,05 [32]. This ICC translates into a cluster size of 25 in a design effect of 2.2.

As such, the definitive sample size for the study would be 3024 patients (1512 in each group) corresponding to 121 basic health-care units (61 in each group, intervention and control).

The sample size calculations were performed with the Granmo program (version 5.2).

### Statistical analysis

Analyses were based on the intention-to-treat principle.

Initially, an analysis of baseline comparability of the groups will be performed with respect to the variables studied. The next phase will be the estimation of raw effect on the outcome variables.

We will use multilevel analyses, with cluster as the random intercept, because of the cluster randomization. Baseline values of the dependent variable will be included as covariates.

We will describe the evolution in health-related quality-of-life (8 scales, and 2 composite measures) in participants over different time periods since quitting smoking. The SPSS statistical package (version 15) and STATA (version 10) are used throughout.

### Quality control

Several procedures were employed to ensure the quality of the study data, thus maximising validity and reliability of the program delivery and outcome assessments. These are:

▪ Written documentation: printed and electronic copies of protocols, session plans and consent forms stored at a central site. All written documentation, including letters sent to participants are standardized across sites and are subject to local institutional ethics committee approval.

▪ Training: telephone interviewers were trained on characteristics and procedures of the study in a 4 hours session.

▪ Regular meetings and mailings between members of the study group (the ISTAPS team) and all participating centers.

▪ Evaluations of teaching sessions: facilitators evaluate the extent of the planned content of each teaching session by completing a standardized evaluation form. This ensures the consistency of the program delivery at each site.

### Ethical aspects

The study respects the Declaration of Helsinki and successive revisions, as well as the norms of good clinical practice. The protocol has been studied and approved by the CEIC – "Comité Ético de Investigación Clínica" (Clinical Investigation Ethical Committee) of the "*Institut d'Investigació Primària Jordi Gol" *[IDIAP-Jordi Gol].

Informed consent: The information was provided verbally as well as in written form to all the participants. The individuals in the study had sufficient opportunity to ask questions regarding the details of the study. The informed consent form conforms to the norms of the Declaration of Helsinki, and as stipulated in the Title I, Article 12 of the (Spanish) Royal Decree 561/1993 of the 16^th ^April, 1993.

Confidentiality of data: Only the investigators and monitors/auditors of the study had access to the data of the subjects who agreed to participate.

## Discussion

The present study evaluates, under near real conditions, the effectiveness in primary care of an overall strategy of smoking cessation, that includes specific interventions addressed to people in all the stages-of-change. It is one of the biggest studies ever undertaken, not only in primary care but at all levels of clinical services. Given the almost universal coverage of primary care in Spain, and considering that many people in the country belongs to social class III to V, it is expected that the majority of the participants of the study will belong to these social classes, generating evidence of effectiveness in this type of population.

Another strength of the study is the large number of health-care professionals and patients who are involved, reinforcing the external validity of the data.

The theoretical framework used is the stages-of-change model of Prochaska and Di Clemente. Despite the fact that some authors such as West [33] or Riensma [12] have questioned the utility of the model in the past years, it remains as one of the most used conceptual frameworks. Since one of the criticism made of the studies using the model is the use of only one of the constructs (the stages of change), in our study we make use of all of them: stages, processes, self-efficacy and decisional balance; we also validate our Spanish version in a complementary study. Health-care professionals who volunteered to participate in the present study could have been more motivated than the health-care professionals working in other PCCs. If this were to be the case, any resulting bias would be in the direction of more conservative estimates of the effects of the intervention. It has been decided to randomize basic care unit instead of smokers as it would be very difficult for a health-care professional (particularly one who is well trained in applying a specific anti-smoking intervention) to withhold this intervention in accordance with the randomization procedure for specific individuals; this would be especially true in the case of the patient having certain pathologies related with smoking or those who had tried to quit many times before, etc. Given the feasibility of recruiting a considerable number of basic care units in control and intervention groups, we believe that this type of cluster randomized assignment is necessary and we hope this will result in comparable groups of basic care unit (health professionals) and patients.

Although the unit of randomization is the basic care unit, the intervention is given at the individual level and the outcomes are also measured at this level. The estimation of the design effect will be taken into acount in all the main analysis.

The ISTAPS study is intended to be a pragmatic trial, trying to develop an intervention that will be applied easily in routine clinical practice. For this reason patients from 14 to 75 years coming to the practice for any reason were included as it is unusual for patients in Spain to consult specifically for smoking cessation advice; in many ocasions these patients presented with comorbidity.

One of the unique roles of primary care teams in smoking cessation and control is to increase the motivation of smokers to quit. In fact, in the many years which elapse between the initiation of smoking (in Spain from 13 to 16 years old) until the beginning of smoking related diseases or other causes of medical consultations, primary care are the only services used by healthy and young or adult population. The ISTAPS study will include more than 60% of their patients in the precontemplation and contemplation stages (approx 1800) and that will allow us to study extensively the effect of motivational interviewing in order to enable an advance in the stage-of-change.

Since the practical clinical guidelines used in the present study have not been yet disseminated, it will enable us to perform the evaluation of the effectiveness using an experimental study design. The guideline will not be available until the end of 2008 and, as such, the study results will not have been influenced by the widespread diffusion of the guidelines.

The availability of health-related quality-of-life data enables us to evaluate the impact of quitting smoking on general health, as well as on specific domains explored by the SF-36 questionnaire. That will be of use when scheduling visits of patients to primary care centres in the post-cessation period, so decreasing the possibility to relapse.

## Conclusion

This study aims to demonstrate the effectiveness of a smoking cessation intervention at the two year post-intervention follow-up. The results will be useful to primary care health professionals in their daily practice.

## Competing interests

The authors declare that they have no competing interests.

## Authors' contributions

CC, CM, JB, SG, CM, EZ, JB, AS and MB form the nucleus of the team of researchers in the ISTAPS project "EFFECTIVENESS, IN PRIMARY HEALTH-CARE CENTERS, OF A QUIT SMOKING INTERVENTION PROGRAM THAT FOLLOWS EVIDENCE-BASED PRACTICAL CLINICAL GUIDELINES". DP took responsibility for the clean up and maintenance of the database as well as processing the manuscript. All authors read and approved the final manuscript.

## Pre-publication history

The pre-publication history for this paper can be accessed here:



## References

[B1] Banegas JR, Diez-Gañan L, Gonzalez-Enriquez J, Villar-Alvarez F, Rodriguez-Artaljo R (2005). La mortalidad atribuible al tabaquismo comienza a descender en España. Med Clin (Barc).

[B2] (2006). Encuesta Nacional de Salud. Ministerio de Sanidad.

[B3] Fiore MC, Jaen CR, Baker TB (2008). Treating Tobacco Use and Dependence: 2008 Update. Clinical Practice Guideline. Rockville, MD: U.S. Department of Health and Human Services. Public Health Service June.

[B4] The Tobacco Use and Dependence Clinical Practice Guideline Panel SaCR (2000). A Clinical Practice Guideline for Treating Tobacco Use and Dependence: A US Public Health Service Report. JAMA.

[B5] West R, McNeill A, Raw M (2000). Smoking cessation guidelines for health professionals: an update. Health Education Authority. Thorax.

[B6] Miller WR, Rollnick S (1999). La entrevista motivacional. Preparar para el cambio de conductas adictivas. Paidós Ibérica. Barcelona.

[B7] Rollnick S, Butler CC, Stott N (1997). Helping smokers make decisions: the enhancement of brief intervention for general medical practice. Patient Educ Couns.

[B8] Butler CC, Rollnick S, Cohen D, Bachmann M, Rusell I, Stott N (1999). Motivational consulting versus brief advice for smokers in general practice: a randomized trial. J Gen Pract.

[B9] Rubak S, Sandbaek A, Lauritzen T, Christensen B (2005). Motivational interviewing: a systematic review and meta-analysis. Br J Gen Pract.

[B10] van Sluijs EMF, van Poppel MNM, van Mechelen W (2004). Stage-based lifestyle interventions in primary care – Are they effective?. American Journal of Preventive Medicine.

[B11] Bridle C, Riemsma RP, Pattenden J, Sowden AJ, Mather L, Watt IS (2005). Systematic review of the effectiveness of health behavior interventions based on the transtheoretical model. Psychology & Health.

[B12] Riemsma RP, Pattenden J, Bridle C, Sowden AJ, Mather L, Watt IS (2003). Systematic review of the effectiveness of stage based interventions to promote smoking cessation. BMJ.

[B13] Rice VH, Stead LF (2000). Nursing interventions for smoking cessation. Cochrane Database Syst Rev.

[B14] Hughes JR, Stead LF, Lancaster T (2007). Antidepressants for smoking cessation. Cochrane Database Syst Rev.

[B15] Prochaska JO, DiClemente CC, Norcross JC (1992). In search of how people change. Applications to addictive behaviors. Am Psychol.

[B16] Prochaska JO, Velicer WF, DiClemente CC, Fava J (1988). Measuring processes of change: applications to the cessation of smoking. J Consult Clin Psychol.

[B17] Pardell H, Jane M, Sanchez I, Villalbi JR, Salto E (2002). Manejo del fumador en la clínica. Recomendaciones para el médico español. Organización Médica Colegial. Madrid/Medicina STM Editores (Ars Médica). Barcelona.

[B18] Russell MA, Wilson C, Taylor C, Baker CD (1979). Effect of general practitioners' advice against smoking. Br Med J.

[B19] Nebot M, Soler M, Martin C, Birules M, Oller M, Sala E (1989). Efectividad del consejo médico para dejar de fumar: evaluación del impacto al año de la intervención. Rev Cli Española.

[B20] Nebot M, Cabezas C (1992). Does nurse counseling or offer of nicotine gum improve the effectiveness of physician smoking-cessation advice?. Fam Pract Res J.

[B21] Quilez C, Hernando L, Rubio A, Estruch J, Fornes MV (1993). Tratamiento del tabaquismo, con chicle de nicotina, en atención primaria. Estudio a doble ciego. Rev Cli Española.

[B22] Salvador LT, Marin TD, Gonzalez QJ, Iniesta TC, Castellvi BE, Muriana SC (1988). [Treatment of smoking: efficacy of the use of nicotine chewing gum. Double-blind study]. Med Clin (Barc).

[B23] Comas A, Suarez R, Lopez ML, Cueto A (1996). Efectividad a largo plazo del consejo antitabaco en Atención Primaria: el proceso de recaída. Rev Esp Salud Publica.

[B24] Torrecilla M, Barrueco M, Maderuelo JA, Jimenez Ruiz CA, Plaza MD, Hernandez MA (2001). Deshabituación tabáquica en la consulta de Atención Primaria: eficacia del consejo médico, la intervención mínima y la terapia sustitutiva con nicotina al año de seguimiento. Aten Primaria.

[B25] Solberg LI, Kottke TE (1988). Doctors helping smokers. A new way to intervene. Minn Med.

[B26] Kottke TE, Brekke ML, Solberg LI, Hughes JR (1989). A randomized trial to increase smoking intervention by physicians. Doctors Helping Smokers, Round I. JAMA.

[B27] Grandes G, Cortada JM, Arrazola A (2001). ¿Podemos ayudar a nuestros pacientes a dejar de fumar? La experiencia del Programa de Abandono del Tabaco. Gac Sanit.

[B28] Martin C, Cordoba R, Jane C, Nebot M, Galan S, Aliaga M (1997). Evaluación a medio plazo de un programa de ayuda a los fumadores. Med Clin (Barc).

[B29] Grandes G, Cortada JM, Arrazola A (2000). An evidence-based programme for smoking cessation: effectiveness in routine general practice. Br J Gen Pract.

[B30] Lyons RA, Lo SV, Littlepage BNC (1994). Perception of Health amongst ever-smokers and never-smokers: a comparison using the SF-36 Health Survey Questionnaire. Tob Control.

[B31] Alonso J, Prieto L, Anto JM (1995). La versión española del SF-36 Health Survey (Cuestionario de salud SF-36): un instrumento para la medida de los resultados clínicos. Med Clin (Barc).

[B32] Campbell MK, Thomson S, Ramsay CR, MacLennan GS, Grimshaw JM (2004). Sample size calculator for cluster randomized trials. Computers in Biology and Medicine.

[B33] West R (2005). Time for a change: putting the Transtheoretical (Stages of Change) Model to rest. Addiction.

